# The CHOICES Black Midwifery Fellowship: An Innovative Model for Training Midwives in Reproductive Justice and Community‐Centered Care

**DOI:** 10.1111/jmwh.70028

**Published:** 2025-09-24

**Authors:** Nikia D. Grayson, Nicole Quinones, Kemetra King, Kiara Norman, Talita Wells Oseguera, Nekea Smith, Miajenell Peake, Alexis Dunn Amore

**Affiliations:** ^1^ CHOICES: Center for Reproductive Health Memphis Tennessee; ^2^ University of California, San Francisco San Francisco California; ^3^ Division of Health Policy and Management, School of Public Health University of Minnesota Minneapolis Minnesota; ^4^ Planned Parenthood Federation of America New York City New York; ^5^ Rory Meyers College of Nursing New York University New York City New York

**Keywords:** Black midwifery, culturally relevant care, midwifery education, midwifery fellowship, postgraduate training

## Abstract

The CHOICES Black Midwifery Fellowship Program is an exemplar of a transformative postgraduate model in midwifery education that extends beyond traditional training frameworks. The program is designed around 5 core pillars: full‐scope reproductive health services education and training, Black feminist thought, leadership and mentorship, neonatal and postnatal care, and community and patient‐centered care. By embedding these frameworks into the curriculum, the fellowship challenges the prevailing medicalized approach to birth that often neglects the unique needs of marginalized communities. This perspective equips fellows with a critical understanding of the social, political, and economic factors that influence health outcomes, enabling them to advocate effectively for their patients and communities.

In addition to improving clinical outcomes, the fellowship has also played a crucial role in restoring trust between Black birthing people and the health care system. Historical injustices and ongoing systemic racism have led to deep‐seated mistrust of mainstream health care institutions among many Black communities. By training Black midwives who understand and share the cultural and lived experiences of their patients, the CHOICES fellowship helps to bridge this trust gap. Fellows are taught to adopt a patient‐centered approach that prioritizes informed consent, shared‐decision‐making, and respect for cultural practices. In this article we outline the structure, curriculum, and training activities of the CHOICES Black Midwifery Fellowship. Additionally, we review the challenges encountered and lessons learned during the implementation process.

## INTRODUCTION

A well‐trained and diverse health care workforce is essential for addressing the persistent disparities in maternal and infant health outcomes in the United States.^1^, ^2^, ^3^ As reproductive health care becomes increasingly complex, the need for comprehensive, culturally competent care that centers the needs of marginalized communities has never been more urgent.^4^ Despite the clear benefits of midwifery‐led care,^4^ a significant shortage of Black midwives continues to limit access to high‐quality reproductive health care for Black birthing people and other underserved populations.^2^, ^3^ This shortage is compounded by systemic barriers, including limited access to midwifery education programs, clinical training sites, and financial barriers such as high tuition costs, insufficient scholarships, and insufficient financial assistance.^5^, ^6^ Aspiring Black midwives face unique challenges due to structural racism such as a lack of mentorship from other midwives of color with similar racial and ethnic identities as them, discrimination in securing professional advancement opportunities and clinical apprenticeships, and a lack of support in midwifery programs for midwifery students of color.^6^

  

Continuing education (CE) is available for this article. To obtain CE online, please visit http://www.jmwhce.org. A CE form that includes the test questions is available in the print edition of this issue.

The CHOICES Black Midwifery Fellowship Program was developed to directly address these gaps through a comprehensive and equity‐focused approach to workforce development. This fellowship not only aims to increase the number of Black midwives but also to equip them as full‐scope reproductive health care providers with the skills needed to provide evidence‐based, culturally responsive care in community‐based settings (eg, birth center, home birth, community clinics). Grounded in the principles of Black feminism and reproductive justice, the program prepares midwives to advocate for and provide care that prioritizes bodily autonomy, equity, and dignity for all patients. The path to becoming a midwife is fraught with barriers, including limited financial resources, access to preceptors, and education programs that lack racial diversity.^6^ The fellowship addresses these challenges by offering financial support and prioritizing diversity in its mentorship and clinical training programs.

**QUICK POINTS**

✦ Despite the clear benefits of midwifery‐led care, a significant shortage of Black midwives continues to limit access to high‐quality reproductive health care for Black birthing people and other underserved populations.
✦ The CHOICES Black Midwifery Fellowship Program provides a template for the development of transformative postgraduate models in midwifery education that extend beyond traditional training frameworks by incorporating Black feminist and reproductive justice frameworks, community‐based learning, and comprehensive reproductive health skills building.
✦ Health care leaders and policymakers must support the expansion of Black midwifery and other racially concordant programs to combat racial disparities in maternal health outcomes.

The fellowship is an extension of the model of care underpinning the clinical services and initiatives at CHOICES: Center for Reproductive Health in Memphis, Tennessee.^7^ CHOICES is an independent, nonprofit comprehensive reproductive health care clinic that provides the full spectrum of reproductive health care services. Established in 1974 one year after the *Roe v. Wade* US Supreme Court decision, CHOICES originally opened as a feminist health care center offering abortions and evolved to offer comprehensive reproductive health care services such as gender‐affirming hormone therapy, sexually transmitted infection testing and treatment, HIV testing, prevention, and referrals, contraception, abortion, prenatal care, and birth services. CHOICES serves a predominantly Black and underserved population, and most patients are insured by Medicaid. In 2020, CHOICES opened the city's first freestanding birth center. A full description of the CHOICES model of care is published elsewhere.^7^

This article outlines the structure and training activities of the CHOICES Black Midwifery Fellowship, highlighting its 5 foundational pillars designed to develop a workforce of midwives who are both clinically proficient and committed to advancing health equity. By examining the challenges and opportunities in diversifying the midwifery workforce, we aim to demonstrate how this fellowship model can serve as a blueprint for nationwide efforts to expand access to comprehensive reproductive health care and improve maternal health outcomes for Black communities and beyond.

## THE CHOICES BLACK MIDWIFERY FELLOWSHIP PROGRAM

The CHOICES Black Midwifery Fellowship was inspired by the urgent need to increase the number of Black midwives in the United States. As of 2024, Black Certified Nurse Midwives (CNMs) and certified midwives represent only 8.3% of the midwifery workforce.^8^ This shortage is rooted in historical injustices that led to the decline of Black midwives, dating back to the 1920s when increased regulations and racial smear campaigns significantly hindered their practice.^9^, ^10^, ^11^ Although the number of Black midwives is slowly increasing, significant barriers must be overcome to address training and workforce issues both for midwifery as a field more broadly and specifically the unique issues that Black midwives in training face.^12^

### Fellowship Development

The CHOICES Black Midwifery Fellowship was developed in response to the urgent need for more Black midwives and culturally responsive health care in the US maternity care system. Grounded in the principles of Black feminist thought and reproductive justice, the fellowship was designed to provide full‐scope clinical training within a supportive, community‐based model. The fellowship is housed at CHOICES in Memphis, which provides a real‐world, community‐based learning environment in which fellows practice across the reproductive health care continuum. Fellows learn how to provide health care in a variety of settings including freestanding birth centers, home births, and practice collaborating with Maternal Fetal Medicine clinicians if it is required for a patient's care. Fellows are trained in such a way that they will be prepared to practice in a variety of clinical settings.

Recruitment for the CHOICES Black Midwifery Fellowship is conducted through multiple channels to ensure a broad and inclusive applicant pool. Announcements are posted on the CHOICES website, shared with midwifery education programs across the country, and distributed through national midwifery listservs and networks. This outreach strategy is designed to reach recent midwifery graduates who are committed to advancing health equity and reproductive justice. All applicants who apply are invited to participate in a first‐round group interview, which helps assess interpersonal skills, alignment with the program's mission, and collaborative potential. Candidates who advance past the first round are invited to the clinic for a second round, in‐person interview, where they engage with staff, tour the clinic and birth center, and learn more about the program. One to 3 fellows are accepted per cycle, and a total of 6 fellows have participated in the program.

The fellowship is a full‐time, 12‐month paid training program. Fellows receive an annual salary of $85,000 and a relocation stipend to support their move. Funding for the fellowship has come from major philanthropic organizations, including the W.K. Kellogg Foundation, Skyline Foundation, and other large foundations committed to health equity. These resources have made it possible to pay fellows a competitive salary, provide relocation support, and build a robust mentorship infrastructure. The program provides intensive training in full‐scope reproductive health care, including prenatal, intrapartum, postpartum, and primary health care, as well as gender‐affirming care, family planning, and abortion education. Each fellow is paired with one or more of the 4 experienced senior midwife mentors on staff and receives structured, ongoing mentorship through monthly meetings and quarterly performance evaluations. The fellowship is designed to create a supportive transition from student to independent practitioner, while centering culturally competent, justice‐oriented care for Black and underserved communities.

### Theoretical Framework

**Figure 1:**
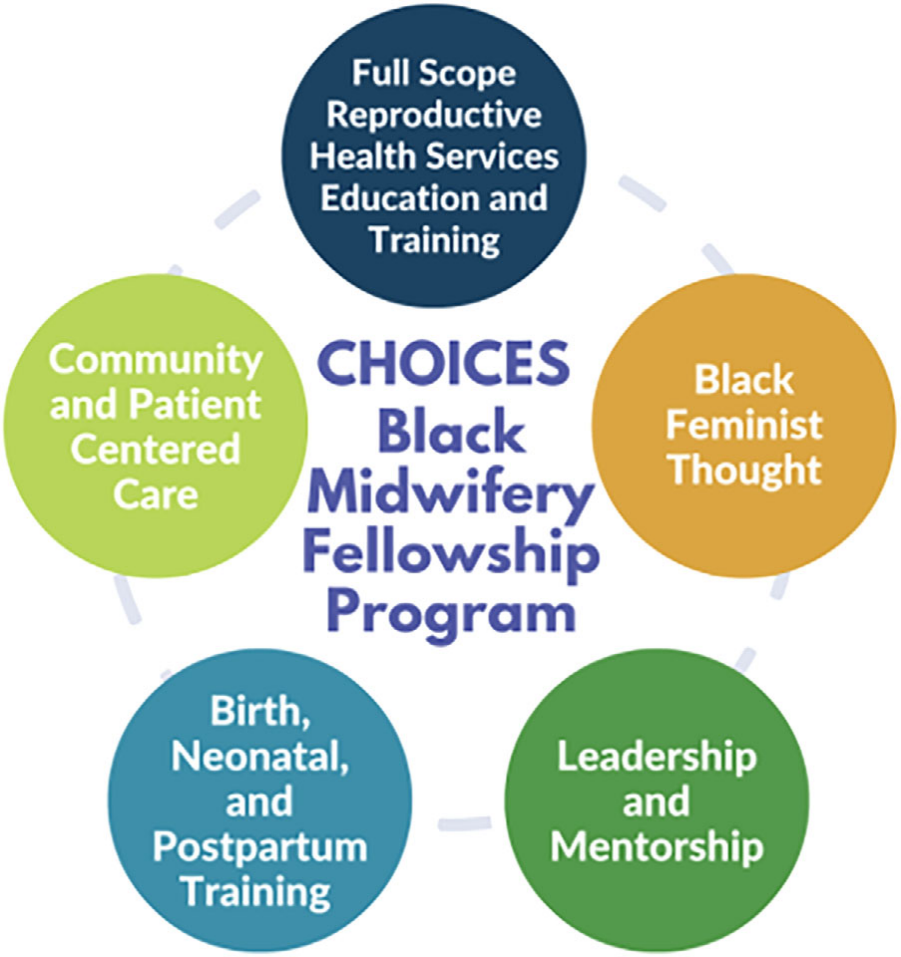
The Five Pillars of the CHOICES Black Midwifery Fellowship Program This framework highlights the 5 core pillars that define the CHOICES Black Midwifery Fellowship Program. Each pillar is designed to equip midwives with the skills, knowledge, and advocacy tools necessary to provide culturally competent and justice‐centered care to marginalized communities.

The CHOICES Black Midwifery Fellowship Program is designed around 5 core pillars: full‐scope reproductive health services education and training, Black feminist thought, leadership and mentorship, neonatal and postnatal care, and community and patient‐centered care (Figure 1). Each pillar plays a crucial role in shaping competent, confident, and socially conscious midwives equipped to address the unique challenges faced by marginalized communities, particularly Black birthing people (Table 1).

**Table 1:** Fellowship Pillars and their Aims

Pillar	Definition	Skills	Issue Addressed
Full‐scope reproductive health services education and training	Ensures that fellows gain comprehensive knowledge and clinical skills necessary for providing high‐quality care across the entire reproductive health spectrum.	Prenatal, intrapartum, postpartum, and primary care, as well as sexual and reproductive health services (and abortion).	By offering such a wide‐ranging learning opportunity, the fellowship addresses a significant gap in traditional US midwifery education when compared with international midwifery standards, which trains midwives to provide comprehensive sexual and reproductive health services in both hospital and community‐based settings (eg, birth center, home birth) and in systems where midwives are integrated into the formal health care system.^13^ This pillar prepares fellows to meet the diverse needs of marginalized populations who face barriers to accessing comprehensive reproductive health care.
Black feminist thought	Black feminist thought is a critical framework used to understand the diverse experiences and intersectional oppressions of Black women^14^ and gender diverse people. The purpose of Black feminist thought is to advocate and ultimately eliminate social injustices and oppression that will improve the lives of all marginalized people and communities.^14^	Fellows expand their understanding of health equity and their clinical practice by incorporating this framework that critically examines the intersections of race, gender, and class within health care systems.^15^	This theoretical foundation empowers fellows to challenge reproductive oppression and advocate for social justice in their practice. By incorporating Black feminist thought, the fellowship not only prepares midwives to provide culturally competent care but also positions them as advocates for systemic change.^14^, ^16^ This pillar aligns the fellowship with the principles of reproductive justice, which seek to ensure that all individuals have the right to have children, not have children, and parent in safe and supportive environments.^17^, ^18^, ^19^
Leadership and mentorship	Focuses on cultivating leadership skills among fellows and providing them with continuous mentorship from experienced midwives and health care leaders.^20^	Upon joining the program, fellows are paired with clinical midwife mentors on staff at CHOICES, who guide them through clinical decision‐making, leadership development, and community engagement. Fellows are also encouraged to identify their strengths, areas for improvement, and set measurable goals that are reviewed quarterly. Additionally, these mentorship‐mentee pairs are racially concordant since all CHOICES midwives on staff are Black identifying.	This structured mentorship is crucial for building confidence, clinical competence, and leadership skills in new midwives, ensuring they are well‐prepared to lead and advocate within their communities.^21^ Pairing Black fellows with Black midwives addresses the gaps in racially and ethnically specific mentorship for midwives of color. By addressing the often overwhelming transition from training to independent practice, this pillar helps reduce burnout and increases retention rates among midwives.^22^, ^23^, ^24^
Neonatal and postnatal care	The focus on neonatal and postnatal care is particularly important for addressing the high infant mortality rates among Black communities in Tennessee.^25^	Fellows receive specialized training in lactation support, infant care, postpartum mental health, and family‐centered care, which are often deemphasized in hospital‐based midwifery education.^26^, ^27^	By equipping fellows with skills to provide compassionate and evidence‐based care during the postpartum period, this pillar directly supports improved maternal and infant health outcomes. Furthermore, it reinforces the role of midwives as primary health care providers who provide continuous and comprehensive care beyond childbirth.
Community and patient‐centered care	Underscores the importance of building trust and partnerships within the communities CHOICES serves by teaching fellows how to provide care in community‐based settings such as a freestanding birth center. This pillar encourages fellows to adopt a community‐based approach to care, which involves active listening, respecting cultural practices, and engaging community stakeholders in health care decisions.^28^ These specific skills have been suggested as ways to improve the experiences of birthing people of color.^28^, ^29^, ^30^	Fellows are trained to engage in policy advocacy to expand access to midwifery care and address the social determinants of health that perpetuate racial disparities and medical distrust.	By integrating reproductive justice principles into patient care, this approach ensures that services are accessible, equitable, and respectful of the unique needs of each patient. This pillar is essential because it tackles the historical mistrust of health care systems within Black communities and strengthens the fellowship's mission to reclaim dignity and autonomy for birthing people of color.^31^

Collectively, these 5 pillars create a robust framework that not only prepares midwives to provide high‐quality, culturally competent care but also empowers them to act as advocates for reproductive justice and systemic change. By prioritizing comprehensive education, mentorship, cultural competence, and community engagement, the CHOICES Black Midwifery Fellowship Program is an exemplar for postgraduate midwifery education that addresses both the immediate health care needs of marginalized communities and the structural barriers that perpetuate disparities in maternal and infant health outcomes.

## INNOVATIVE APPROACHES IN MIDWIFERY EDUCATION

The CHOICES Black Midwifery Fellowship Program provides a template for the development of transformative postgraduate models in midwifery education that extend beyond traditional training frameworks. In Table 2, we outline activities that align with the core pillars of the CHOICES fellowship program. The activities are implemented according to the learning needs of the fellow and their requested timeline to achieve competency related to clinical skills and experiences. Additionally, the activities are carried out within a context of community and support.

**Table 2:** CHOICES Black Midwifery Fellowship Program Activities

Core Pillar	Example of Activities Implemented
Full‐scope reproductive health services education and training	Clinical training in out‐of‐hospital birth. Training in gynecology clinic for routine and complex gynecologic care. Gender‐affirming care training to provide inclusive and respectful care for LGBTQ+ patients. Family planning services training, including contraceptive counseling and management, and all options counseling. Postabortion care.
Black feminist thought	Journal Club Discussions on Black Feminist Theory. Critical readings and discussions of texts such as *Killing the Black Body* by Dorothy Roberts, *Policing the Womb* by Michele Goodwin, and *Medical Apartheid* by Harriet A. Washington Seminars on reproductive justice.
Neonatal and postnatal care	Hands‐on training and certifications: Neonatal Resuscitation Program (NRP), Breastfeeding support workshops, Spinning Babies workshops for fetal positioning management, and Birth Emergency Skills Training (BEST). Postpartum home visits for newborn assessments, lactation support, and mood disorder assessment. Clinical training at birth center.
Leadership and mentorship	Structured mentorship model pairing new midwives with senior midwives. Monthly check‐ins for progress review and goal refinement.
Community and patient‐centered care	Community‐based training through visits to: home birth practices, other birth centers and Mamatoto Village for holistic family support. Attendance at conferences: Black Mamas Matter Alliance Maternal Health Conference, Alabama Black Midwives Conference, ACNM Annual Conference, Society of Family Planning Annual Meeting, National Abortion Federation Conference, and Abortion Care Network Conference. Patient‐centered communication training for informed consent and shared‐decision‐making.

Abbreviations: ACNM, American College of Nurse‐Midwives; LGBTQ+, lesbian, gay, bisexual, transgender, and queer.

### Abortion Training Exemplar

Although a variety of training opportunities are woven throughout the curriculum of the fellowship, one of the most groundbreaking and critical areas of training is in abortion care. Historically, midwifery programs have lacked comprehensive abortion and miscarriage management training because of restrictive state scope of practice policies, institutional limitations, and prevailing antiabortion sentiments.^32^, ^33^, ^34^, ^35^ This gap in education and clinical exposure has left many midwives underprepared to provide essential reproductive health care services, despite the reality that abortion care is an integral part of comprehensive sexual and reproductive health. Recognizing this deficiency, the fellowship was intentionally designed to equip Black midwives with the knowledge, skills, and clinical competencies necessary to provide abortion care.

Beyond the technical aspects of training, the inclusion of abortion care in the fellowship aligns with the principles of reproductive justice.^17^, ^18^, ^19^ By centering abortion care within the fellowship, this program ensures that Black midwives are not only skilled providers but also empowered advocates who can address health care inequities and fight for policies that support bodily autonomy and reproductive freedom.

The fellowship welcomed its first cohort of recently graduated midwives in the summer of 2022, a pivotal moment in reproductive health history. Just weeks after their training began, the Supreme Court issued its decision in *Dobbs v. Jackson Women's Health Organization*, overturning *Roe v. Wade* and eliminating the federal constitutional protection for abortion. This seismic policy shift returned oversight of abortion regulation to individual states, leading to an immediate wave of restrictive laws and clinic closures across the country. In Tennessee, where the fellowship is based, this ruling had an especially devastating impact. In August 2022, the state enacted a total abortion ban, effectively criminalizing nearly all abortion care. This law not only stripped patients of their reproductive autonomy but also significantly altered our ability to provide abortion care and abortion training for our fellows.

With Tennessee's total ban in place, the fellows faced significant limitations in their ability to gain hands‐on clinical experience in abortion management. Despite these barriers, the fellowship remained steadfast in its commitment to ensuring that abortion care remained a core component of the curriculum. Fellows engaged in rigorous discussions on abortion care protocols, patient‐centered counseling, legal considerations, and strategies for navigating situations after the *Dobbs* decision, ensuring that they remained well‐informed and prepared to integrate this critical service into their practice where legally permissible. By training Black midwives in abortion care, even in the face of legal and political challenges, the fellowship underscores the fundamental role of midwives in safeguarding reproductive autonomy.

### Community and Support

In addition to the core activities outlined in the training plan, the learning experience is grounded within a learning environment of support. The mentorship model of the fellowship acts as a community component of the program, designed to bridge the gap between academic learning and real‐world clinical practice. Upon entry into the program, fellows self‐reflect to identify their strengths and areas for growth, set measurable goals, and receive individualized mentorship from CHOICES midwives to help them achieve these goals. Given that the retention of midwives in community birth settings can be challenging,^36^ the fellowship program was designed with a 6‐month transition period to promote hands‐on mentoring and allows the fellow time to build confidence before practicing independently.

Regular feedback sessions with mentors allow fellows to reflect on their practice, build confidence, and enhance their clinical decision‐making skills. Additionally, fellows were taken to a variety of cities including Atlanta, Washington, DC, and other areas to visit and shadow midwives in a variety of practice settings. This structured mentorship is particularly valuable given the challenges new midwives face in transitioning to independent practice, and it addresses a common gap in traditional midwifery education programs that often lack robust support systems for recent graduates.^23^, ^24^

## IMPACT ON MATERNAL HEALTH EQUITY

The CHOICES Black Midwifery Fellowship Program was designed to address racial disparities in maternal and infant health outcomes by training midwives to provide culturally competent and justice‐centered care. Additionally, the program emphasis on racially concordant care is a key focus, which research shows can significantly improve patient satisfaction, trust, and outcomes.^37^, ^38^, ^39^, ^40^ The fellowship prioritizes the training of Black midwives to serve communities that share their racial and cultural backgrounds, directly addressing the distrust many Black birthing people feel toward the health care system.^31^ This approach is not only culturally affirming but is also evidence‐based,^37^, ^38^, ^39^ aligning with studies that highlight the positive impact of ethnically matched care on health care usage and outcomes.

By increasing the number of Black midwives serving marginalized communities, the program seeks to reduce maternal mortality rates and improve birth outcomes for Black birthing people^6^ in Tennessee. Research has shown that midwifery‐led care models, particularly those that incorporate culturally congruent care, are associated with lower rates of preterm births, cesarean sections, and other medical interventions.^41^, ^42^, ^43^ The fellowship's emphasis on providing full‐scope reproductive health care, including prenatal, intrapartum, postpartum, and primary care, ensures that birthing people receive continuous and comprehensive health care, which is crucial for preventing complications and promoting positive health outcomes.^44^

### Challenges and Lessons Learned

The implementation of the fellowship program at CHOICES required continuous feedback from the fellows, midwife mentors, and clinical midwife administrators overseeing the program. Meetings were held on a monthly to quarterly basis pending the needs of the fellow in training. During these sessions conversations were held to discuss strengths, areas for improvement, and goals to ensure that experiences were in alignment with training needs. Ensuring an appropriate evaluation of the effectiveness of the program is a necessary component to ensure quality improvement and revision for future participants in the program.

The most common areas of growth reported by fellows across the first 3 cohorts of the program included feeling more confident in their labor management skills particularly in a community birth setting as well as feeling more confident as a health care provider. The group also reported that receiving midwifery training grounded in a more holistic approach helped them to feel more confident in providing education and building rapport with clients. The program encouraged them to develop autonomously as clinicians and overall helped them feel more secure in providing reproductive care services, including perineal health as well as primary care and reproductive wellness services.

The common challenges reported by participants in the program were related to adapting to the pressure of working in a community setting with smaller teams and feeling confident in their clinical skills. Most of the fellows were accustomed to working in hospital settings during their clinical experiences where there were large teams for back up in the case of emergencies. Learning how to manage emergencies in a smaller team setting was hard and required time for confidence to develop. Additionally, fellows reported that they struggled with developing confidence in transitioning from supervision to clinical independence, despite having the 6‐month transition period into practice. This was driven by the desire to become more efficient in triage so that they could more accurately determine when a patient was no longer appropriate for out‐of‐hospital care. Fellows also reported that their academic programs presented a more medicalized training model that made the transition to community birth midwifery more challenging. These findings suggest that fellowship programs grounded in community‐based midwifery are needed to ensure more robust and well‐rounded training of the midwifery workforce.

Finally, another major challenge fellows encountered was obtaining comprehensive abortion training. Although abortion care is a core tenet of the fellowship, restrictive state laws, particularly the total abortion ban enacted in Tennessee in August 2022 following the *Dobbs* decision severely limited direct clinical training opportunities. Fellows expressed frustration over the inability to gain hands‐on abortion training due to legal barriers.

## CONCLUSION

The CHOICES Black Midwifery Fellowship Program serves as a model for advancing maternal health equity through culturally competent and justice‐centered midwifery education. The fellowship program's emphasis on mentorship, reproductive justice, and community‐centered care highlights the need for similar initiatives nationwide. The fellowship incorporates community‐based training that emphasizes the importance of providing care in settings that are accessible and familiar to the communities being served. This model trains fellows to integrate community resources, engage with local stakeholders, and advocate for policy changes that expand access to midwifery care. By training midwives to practice in community‐based settings such as birth centers and home births, the fellowship challenges the dominant hospital‐centric model of birth in the United States.^45^ This not only increases birthing options for marginalized communities but also aligns with evidence suggesting that community‐based midwifery care is associated with lower rates of medical interventions and improved maternal and neonatal outcomes.^46^, ^47^

The presence of a Black‐led midwifery team at CHOICES has been instrumental in increasing access to midwifery care for Black birthing people in Memphis. This approach aligns with evidence suggesting that midwifery‐led care models improve maternal and neonatal outcomes by reducing interventions and increasing patient satisfaction.^48^ Future research should examine the impact of similar mentorship programs on the sustainability of the Black midwifery workforce. Health care leaders and policymakers must support the expansion of Black midwifery programs to combat racial disparities in maternal health outcomes. Investment in such programs is essential to restoring dignity, trust, and justice in birth care for people of color.

## CONFLICT OF INTEREST

The authors have no conflicts of interest to disclose.
